# Homozygosity for the Common IL23R R381Q Variant Associates with Increased Susceptibility to Chronic Mucocutaneous Candidiasis

**DOI:** 10.1002/eji.70002

**Published:** 2025-07-20

**Authors:** Margaux Gerbaux, Frederik Staels, Mathijs Willemsen, Julika Neumann, Leoni Bücken, Lize Van Meerbeeck, Willem Roosens, Adrian Liston, Stéphanie Humblet‐Baron, Rik Schrijvers

**Affiliations:** ^1^ KU Leuven Department of Microbiology, Immunology and Transplantation Adaptive Immunology Laboratory Leuven Belgium; ^2^ KU Leuven Department of Microbiology, Immunology and Transplantation Allergy and Clinical Immunology Research Group Leuven Belgium; ^3^ Pathology Department University of Cambridge Cambridge UK

**Keywords:** chronic mucocutaneous candidiasis, IL23R, Inborn errors of immunity, TH‐17

## Abstract

Chronic mucocutaneous candidiasis can be caused by an Inborn Error of Immunity, especially those affecting TH‐17 / IL‐17 responses. Through in‐depth immunophenotyping and functional assays, we reveal the association between homozygous carriage of the common p.R381Q IL‐23R genetic variant and increased candidiasis susceptibility, relying on disrupted IL‐23‐mediated IL‐17 immunity.

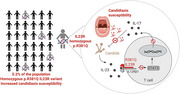

Chronic mucocutaneous candidiasis (CMC) is characterized by recurrent or persistent cutaneous and/or mucosal candidiasis [1]. This condition can reveal an Inborn Error of Immunity (IEI), especially those affecting TH‐17 / IL‐17 responses [1, 2].

IL‐23 binds to the IL23 receptor (IL23R) complex, formed by IL12Rβ1 and IL23R, at the surface of T cells, causing STAT3 phosphorylation, IL‐17 production, and TH‐17 immunity [2]. The recent report of two IL23R‐deficient kindreds with CMC and impaired IL‐17 immunity due to disrupted IL‐23 signaling highlighted its importance for mucosal antifungal responses [3].

IL23R p.R381Q variant is present in heterozygous and homozygous states in 5.48% and 0.18% of the population (gnomAD v4.1.0), respectively. This heterozygous variant has been reported in genome‐wide association studies (GWAS) as being protective against inflammatory diseases and associated with increased severity of *Mycobacterium tuberculosis* infection [4, 5, 6]. Its protective effect was demonstrated to occur through defective response to IL‐23. After IL‐23 stimulation, Hela cells transfected with the R381Q IL23R variant induced significantly lower STAT3 phosphorylation as compared with the wild‐type (WT) allele, while primary cells from R381Q heterozygous carriers produced less IL‐17 as compared with WT healthy controls (HCs) [7, 8]

Here, we studied a homozygous carrier of the R381Q IL23R variant suffering from CMC and investigated the hypothesis of this variant acting as a genetic risk factor for *Candida* infection.

The index patient is a 26‐year‐old female suffering from seven episodes of unprovoked oro‐esophageal candidiasis (Figure 1A), ultimately requiring chronic antimycotic treatment. Vaginal candidiasis repeatedly occurred after antibiotics. She reported no other relevant infection. Routine immunological evaluation did not reveal any striking abnormalities and excluded secondary immunodeficiencies (Table S1). Target panel sequencing for IEI was negative. Whole‐exome sequencing (WES) identified a homozygous missense variant in IL23R (c.1142G>A, p.R381Q, rs11209026) as the sole candidate based on pathogenicity predictions (CADD score 26), rarity in the homozygous state, high conservation across species, and its biological relevance. The R381Q variant resides in the intracellular domain of IL23R, before the JAK2 binding site, necessary for downstream STAT3 signaling (Figure 1B). No other relevant variants were identified (Table S2). The parents were healthy and heterozygous while the 24‐year‐old brother was homozygous for the R381Q variant (Figure 1A) and reported recurrent onychomycosis, tinea pedis, and one episode of genital mycosis.

**FIGURE 1:**
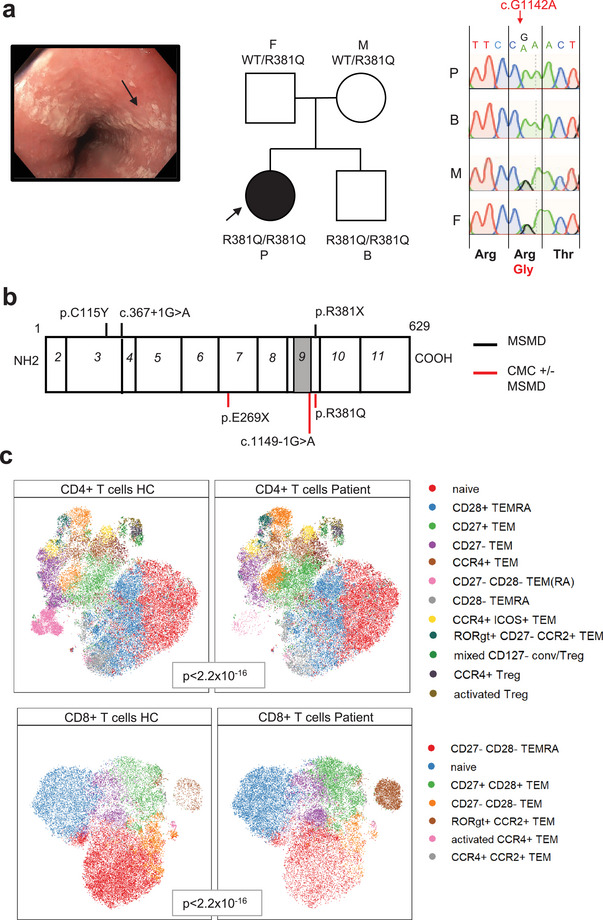
Homozygous R381Q IL23R variant in a patient with CMC. (A) Esophageal candidiasis in the patient. Family genotype. Sanger chromatograms of IL23R sequencing. (B) Linear structure of IL23R protein (extracellular, transmembranous (grey) and intracellular) and exon (italic) with previously reported variants. (C) t‐SNE representations of CD14‐CD3+ CD8+ or CD4+ T cells from HCs (*n* = 3) and patients (two technical replicates) with FlowSOM clustering. Kolmogorov–Smirnov comparisons.

The patient's immune compartment was investigated by flow cytometry (Figure 1C), showing an enrichment of TH‐17‐like (RORgT^+^ CCR2^+^) TEM CD4^+^ and CD8^+^ T cells and a decrease of the terminally activated and differentiated CD8^+^ and CD4^+^ T cells (CD27^−^ CD28^−^ TEM/TEMRA CD8^+^ and CD4^+^ T cells).

The immune response to fungal infection was studied by assessing the response of peripheral blood mononuclear cells (PBMC) to heat‐killed *C. albicans* (HKCA), pro‐TH‐17 cytokines, or the positive control IFNα2b. A significant production of IL‐17 in response to HKCA was observed for the HCs (not genetically tested due to ethical limitations) and the father. In contrast, the patient's cells failed to respond, and the brother and mother responded poorly (Figure 2A). IL‐23 stimulation led to a significant production of IL‐17 from HCs and the father, whereas the patient, her brother, and mother exhibited reduced IL‐17 production (Figures 2B,C). Similarly, lower STAT3 phosphorylation was observed in the patient's T cells in response to IL‐23 (Figure 2D), while the response to other cytokines was conserved (Figure 2B,D).

**FIGURE 2:**
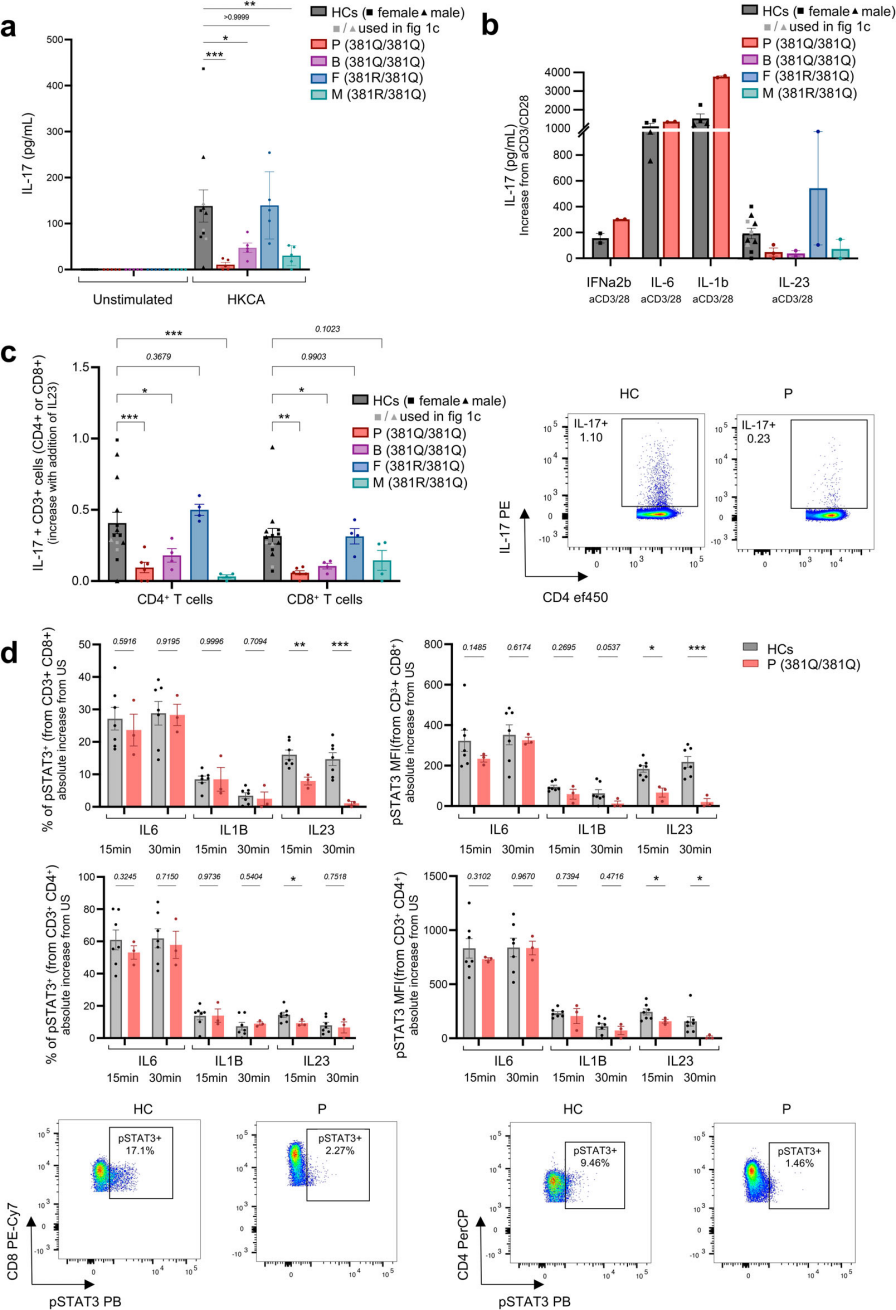
Impaired response to HKCA and IL‐23 in R381Q carriers. IL‐17 production or STAT3 phosphorylation were measured by ELISA (A, B) or flow cytometry (C, D) after stimulating PBMC with HKCA, IL‐23, IL‐1β, IL‐6, or IFNα2b. Each dot represents 1 HC (A) *n* = 11, (B) *n* = 10, (C) *n* = 14, (D) *n* = 7) or one independent replicate. Mean + SEM. Two‐way ANOVA for repeated measures with Sidak's comparison, *p < 0.05, **p < 0.01, ***p < 0.001.

In this report, we reveal, for the first time, the association between homozygous carriage of the hypomorphic loss‐of‐function R381Q IL23R variant and susceptibility to candidiasis. We hypothesize a mechanism based on impaired IL‐23‐mediated STAT3‐dependent IL‐17 production by CD4^+^ and CD8^+^ T cells, leading to a defective response to *Candida*. This defect was observed in the two R381Q homozygous individuals and one of the heterozygous carriers. The homozygous R381Q IL23R variant translated into a clinical susceptibility to Candida with a variable expressivity within this family and with an incomplete penetrance overall, considering its frequency in the public database. This is consistent with previous studies reporting reduced production of IL‐17 in some, but not all, R381Q heterozygous carriers [7, 8]. Studying these defects in Th17 polarizing conditions with the addition or absence of IL‐23 or in MAIT cells would be interesting, considering their importance in CMC [3]. Our immunophenotyping results confirm the ability to generate TH‐17 cells despite IL23R deficiency, consistent with previous reports of IL‐23R deficiency and CMC, and suggest suboptimal activation with a persistence of pro‐TH‐17 stimuli due to a lack of efficient *Candida* clearance [3]. These observations support the predominant role of IL‐23 in the effector function of TH‐17 cells rather than their initial differentiation and it would be interesting to investigate it in *Candida*‐specific setups.

In both IL23R and IL12Rβ1 deficiencies, only 30% of the deficient carriers present with CMC [2]. This might in part result from environmental factors, especially exposure to Candida, or provocative events such as antibiotics, but also supports a redundancy of IL‐17 response, controlled by the interplay of various cytokines, including IL‐23. Interestingly, the onset of the disease occurred at adolescence, and women exhibited a more profound impairment than men, suggesting that gender, especially hormones such as estrogens (known to be a risk factor for candidiasis), could contribute to the penetrance [9]. This should be confirmed in larger setups. The genetic background, including other polymorphisms working synergistically or antagonistically, might also affect the expressivity by affecting the same or another signaling pathway overlapping in immune endpoints. Interestingly, in GWAS, the combination of multiple IL‐23R SNPs in the same subject was even more protective against psoriasis than a single one [4].

GWAS have highlighted the protective effect of the R381Q IL23R variant against IL‐17‐driven diseases, conferred through impaired IL‐23‐mediated TH‐17 responses [4, 5, 6, 7, 8]. Allele conferring protection against a disease by tempering one immune pathway could, by the same mechanism, lead to susceptibility to other diseases. Yet, no association has been made so far between the R381Q IL23R variant and increased susceptibility to *Candida*. We hypothesize that this is due to a lack of investigation in CMC cohorts, or a more subtle phenotype, and that the homozygous R381Q IL23R variant acts as a genetic risk factor for *Candida* infections. Considering the frequency of this variant, further studies should focus on comparing its prevalence in CMC population versus controls in large cohorts. Genetic determinants of common infections might be more frequent than anticipated and represent major genetic risk factors for developing infectious diseases, which is likely to affect a small but not negligible proportion of the population [10]. CMC remains underdiagnosed and often requires long‐term antifungal treatment. Genetic predisposition may highlight a subset of patients requiring specific management, including drugs targeting the dampened immune response rather than the infectious burden, especially considering the widespread prevalence of *Candida* infections and drug resistance [9]. We urge for more prompt genetic investigations as they could guide treatment strategies and lead to major changes in current medical practice.

## Authors Contributions

Margaux Gerbaux and Frederik Staels initiated the study, performed genetic analysis, designed and performed the experiments, and analyzed the data. Margaux Gerbaux drafted the manuscript. Mathijs Willemsen collected human material, helped perform genetic analysis, and contributed to the conceptualization of the experiments. Julika Neumann helped in performing and analyzing the immunophenotyping data and contributed to the conceptualization of the experiments. Leoni Bücken helped to perform genetic analysis and the immunophenotyping. Willem Roosens assisted in performing experiments. Adrian Liston contributed to the conceptualization of the study. Stéphanie Humblet‐Baron and Rik Schrijvers initiated and supervised the study and contributed to the conceptualization of the study. Frederik Staels and Rik Schrijvers were involved in medical care. All authors commented on previous versions of the manuscript, revised and approved the final manuscript.

## Ethics Statement

All experiments were approved by the KU Leuven ethics committee, and written informed consents were obtained from all participants.

## Conflicts of Interest

The authors declare no conflicts of interest.

## Peer Review

The peer review history for this article is available at https://publons.com/publon/10.1002/eji.70002.

## Supporting information




**Supporting Information file 1**: eji70002‐sup‐0001‐SuppMat.pdf

## Data Availability

The data supporting the findings of this study are available from the corresponding author upon reasonable request.
