# Supplementary material for: Homozygosity for the Common IL23R R381Q Variant Associates with Increased Susceptibility to Chronic Mucocutaneous Candidiasis
Source: Eur J Immunol. 2025 Jul 20;55(7):e70002. doi: 10.1002/eji.70002 (PMC12277866; doi:10.1002/eji.70002)

Supplementary material

Homozygosity for the common IL23R R381Q variant associates with increased susceptibility to chronic mucocutaneous candidiasis

Margaux Gerbaux*[1], Frederik Staels*[1,2], Mathijs Willemsen[1], Julika Neumann[1], Leoni Bücken[1], Lize Van Meerbeeck[1], Willem Roosens[2], Adrian Liston[3], Stéphanie Humblet-Baron[†# 1], Rik Schrijvers[†# 2]

1 KU Leuven, Microbiology, Immunology and Transplantation Department, Adaptive Immunology Laboratory, Belgium

2 KU Leuven, Microbiology, Immunology and Transplantation Department, Allergy and Clinical Immunology Research Group, Belgium

3 Pathology Department, University of Cambridge, United Kingdom

*, † equally contributed

# corresponding authors

Methods

Panel target sequencing: Target panel sequencing containing 226 known inborn errors of immunity genes, including validated genes involved in CMC (*STAT1, CARD9, IL17F, IL17RA, IL17RC).*

No genetic testing was performed on healthy controls due to ethical limitations.

gDNA extraction and whole exome sequencing

Purelink DNA Genomic DNA Mini kit (ThermoFisher Scientific) was used according to the manufacturers' instructions for gDNA extraction from whole blood. Whole exome sequencing (WES) was performed using SureSelect Human All Exon V7 (Agilent) for exome capture (Macrogen®, Netherlands).

Whole Exome Sequencing (WES)

Variants within the protein coding or splice regions identified by whole exome sequencing :

- 51 autosomal and 0 X-linked recessive homozygous variants with a frequency below 10%
- 200 and 28 autosomal and X-linked dominant heterozygous variants with a frequency below 0.1%
- 89 and 28 compound heterozygous autosomal and X-linked variants with a frequency below 1%
- complete list available from corresponding author upon reasonable request

All potential variants were investigated and no relevant variant (including existing or new mutations in genes known to be associated with CMC) were identified apart from the homozygous missense variant in *IL23R* (c.1142G>A, p.R381Q, rs11209026).

Peripheral blood mononuclear cells (PBMC) isolation

Peripheral blood mononuclear cells (PBMC) were isolated from whole blood using density gradient centrifugation. Whole blood was diluted in equal volume of RPMI 1640 (Gibco^{TM}) supplemented with 10% FBS and layered over lymphocyte separation medium (LSM) (MP Biomedicals) before separation by centrifugation (400 rcf for 25 min). PBMCs were collected, washed in RPMI and either frozen in FBS with 10%

DMSO before long-term storage in liquid nitrogen or used immediately for functional assays.

PBMC stimulation

Fresh or thawed frozen PBMCs were counted and plated at a density of 1x106 cells/mL (2x105 cells/well) in a 96-well plate in complete RPMI 1640 (GibcoTM) supplemented with penicillin–streptomycin (P/S), 10% FBS, 25 mM HEPES and MEM non-essential amino acids) and rested overnight.

IL-17 measurement after heat-killed Candida albicans (HKCA) stimulation

At day 1 of culture, HKCA (Invivogen, $1x10^7$ cells/mL) was added. Supernatant was collected after 6 days for measurement of IL-17 concentration by ELISA.

IL-17 measurement after IL-1b, IL6, or Interferon-a2b IL-23 stimulation

At day 1 of culture, cells were transferred onto an anti-CD3 (10 µg/mL) pre-coated 96-well plate with addition of soluble anti-CD28 (5 µg/mL), IL-23 (R&D, 100 ng/mL), IL-1β (Biolegend, 20 ng/mL), IL-6 (Miltenyi, 25 ng/mL) or Interferon α2b (IFNα2b) (R&D, 10 ng/mL). At day 4, Brefeldin A (Abcam, 5 µg/mL) was added for 6 hours to the culture to assess intracellular cytokine production by flow cytometry and the supernatant was collected. Gating strategy depicted in Supplementary Figure S2.

STAT3 phosphorylation following IL-23, IL-1b, IL-6 or Interferon-a2b stimulation

On day 2 of culture, cells were serum-starved for 4 hours, before stimulation with IL-23 (R&D, 20 ng/mL), IL-1b (Biolegend, 20 ng/mL), IL-6 (Miltenyi, 25 ng/mL) or Interferon a2b (IFNa2b) (R&D, 10 ng/mL) for 15 to 30 minutes. The phosphorylation of STAT3 was assessed by flow cytometry. Gating strategy depicted in Supplementary Figure S3.

Staining

Cells were washed in PBS and plated in a 96-well V-bottom plate before staining. Data acquisition was performed on the BD FACSCanto II or the Sony ID7000 Spectral analyzer and analyzed with FlowJo software.

For IL-17 measurement: Cells were stained with anti-CD4 (ef450, Invitrogen), anti-CD8 (PE-Cy7, Invitrogen) and zombie aqua (ZA) fixable viability dye for 30 minutes at RT. After washing with FACS buffer, cells were fixed (eBioscience Foxp3 Fix/Perm), permeabilized (eBioscience) and stained intracellularily: anti IL-17 (PE, ThermoFisher Scientific) for 30 min at RT.

For STAT3 phosphorylation: Cells were immediately fixed in 2% paraformaldehyde for 30 minutes at RT, washed twice in PBS, and permeabilized with 100% ice-cold methanol for 20 minutes on ice. Subsequently, cells were stained for phospho-STAT3 (Pacific Blue, BD) overnight at 4°C. The following day, cells were washed once in PBS before staining for CD3 (FITC, eBioscience), CD4 (PerCP, Biolegend) and CD8 (PE-Cy7, Invitrogen) for 1h at RT.

For the immune phenotyping: PBMC were plated at a density of 8x10^6 cells/well, washed with PBS, blocked with True-stain blocker solution (BioLegend) and washed with FACS buffer before staining for 1 hour at 4°C with surface markers: anti-CD8-BUV805, anti-CD4-BUV496, anti-CXCR3-BV785, anti-CCR2-BV605 (BD Biosciences), anti-CD3-PerCP-Vio700 (Miltenyi Biotec), anti-CD45RA-FITC, anti-CD14-PE-Cy5.5, anti-CCR7-PE-Cy7 (eBioscience), anti-CD25-BV711, anti-CCR4-PE/Dazzle 594 (BioLegend). Cells were then washed with PBS before staining with Fixable viability dye eFluor780 (eBioscience™) and further washed with FACS buffer. After fixation (eBioscience Foxp3 Fix/Perm) and permeabilization (eBioscience), cells were stained overnight with intracellular markers: anti-RORγt-PE (BD Biosciences) and anti-FOXP3-AF647 (Bio Legend) and washed in FACS buffer.

Immune phenotyping analysis

Unsupervised analysis of flow cytometry data of PBMC from HCs and patient was performed using t-SNE representation and FlowSOM clustering with clusters called according to their expression of subtype-specific markers, as previously described (1-2). Equal numbers of events were used for each condition to calculate the t-SNE representation, avoiding clustering bias based on the number of individuals per group. Lineage markers (CD14, CD3, CD4, CD8, FoxP3, CD45RA, CCR7) were used to cluster major leukocytes subsets with conventional method of FlowJo software, before analyzing subsets of total $CD4^+$ T cells (FoxP3, CD45RA, CCR7, CCR4, RORgT, CD27, CXCR5, ICOS, CD28, Ki67, CD95, CD127, HLA-DR, CCR2,

CTLA-4) and CD8+ T cells (CD45RA, CCR7, CCR4, RORgT, CD27, CXCR5, ICOS, CD28, Ki67, CD95, CD127, HLA-DR, CCR2, CTLA-4). The identity of each cluster was determined based on histograms showing the density distribution per cluster as shown in Supplementary Figure S1. Comparisons between HCs and the patient were performed using Kolmogorov-Smirnov comparison tests of the cross-entropy distributions of the t-SNE representations as described in Roca et al. (1)

ELISA

The concentration of IL-17 in the supernatant was measured by ELISA according to the manufacturer’s instructions (R&D).

Statistical analysis

All statistical analysis were carried out using GraphPad Prism (GraphPad Software). Statistical analysis was only performed if at least three technical replicates were available. Ordinary Two-way ANOVA for repeated measures with Sidak’s multiple comparison test was applied to compare multiple groups with repeated measures over time, used to compare HCs to P, B, F and M.

All significance levels were defined as followed: *$p < 0.05$, **$p < 0.01$, ***$p < 0.001$

The statistical significance between t-SNE representations between HCs and P was calculated based on Kolmogorov-Smirnov comparisons.

Authors contributions*:* MG and FS initiated the study, performed genetic analysis, designed and performed the experiments, analyzed the data and drafted the manuscript. MW collected human material, helped performing genetic analysis and contributed to the conceptualization of the experiments. JN helped performing and analyzing the immune phenotyping and contributed to the conceptualization of the experiments. LB helped performing genetic analysis and the immunophenotyping. EVH and WR assisted in performing experiments. AL contributed to the conceptualization of the study. SHB and RS initiated and supervised the study and contributed to the conceptualization of the study. FS and RS were involved in medical care. All authors commented on previous versions of the manuscript, revised and approved the final manuscript.

Ethics approval and consent to participate*:* All experiments were approved by the University of Leuven ethics committee (S58466) and conducted according to the University of Leuven ethics guidelines and the principles of the Helsinski Declaration. Written informed consent was obtained from patients and healthy controls subjects.

Consent to publish: Written informed consent for the publication of this research was obtained from all study participants.

Supplementary references

1. Roca CP, Burton OT, Neumann J, Tareen S, Whyte CE, Gergelits V, et al. A cross-entropy test allows quantitative statistical comparison of t-SNE and UMAP representations. Cell Rep Methods. 2023 Jan 23;3(1):100390.

2. Canti L, Humblet-Baron S, Desombere I, Neumann J, Pannus P, Heyndrickx L, et al. Predictors of neutralizing antibody response to BNT162b2 vaccination in allogeneic hematopoietic stem cell transplant recipients. J Hematol OncolJ Hematol Oncol. 2021 Oct 24;14(1):174.

Supplementary Table S1: Routine immunological evaluation of the index patient. Candida, PHA and STAT1-index represent the fold change for the respective condition compared to the unstimulated (medium) condition.

**Laboratory evaluation**	**Value**	**Reference value**
Hemoglobin (g/dL)	13.3	12.0-16.0
Thrombocytes (x10^9/L)	263	150-400
Leukocytes (x10^9/L)	6.96	4.0-10.0
Neutrophils (x10^9/L)	3.8	2.5-7.8
Lymphocytes (x10^9/L)	2.44	1.208 – 3.586
B cells ($CD19^+$) (x10^9/L)	0.238	0.082-0.476
IgG (g/L)	**6.84**	7.51-15.6
IgA (g/L)	1.54	0.82-4.53
IgM (g/L)	1.06	0.46-3.04
T cells ($CD3^+$) (x10^9/L)	2.045	0.798-2.823
$CD4^+$ T cells	1.407	0.455-1.885
$CD8^+$ T cells	0.684	0.219-1.124
NK cells ($CD3^-$ $CD56^+$)	0.170	0.066-0.745
Lymphocyte stimulation test<br>Candida – index x10^3 cpm<br>PHA – index x10^3 cpm	 14.46<br>876	≥ 5
STAT-1 phosphorylation<br>IFN-α - index<br>IFN-γ - index	 2.02<br>2.5	 2.44 (HC n=1)<br>3.7 (HC n=1)

Supplementary Table S2A: List of all homozygous variants (n=50) with allelic frequency of < 0.10 identified in the index patient (single-index WES).

Chr	Position	Gene	Location	Ref	Alt
1	16730307	SPATA21	Exon	G	T
1	16865769	FAM231B	Exon	CT	C
1	57415309	C8B	Exon	G	A
1	57422510	C8B	Exon	C	T
1	67705957	**IL23R**	Exon	G	A
1	148004732	NBPF14	Exon	T	C
1	152185811	HRNR	Exon	C	CAG
1	152185816	HRNR	Exon	CCA	C
1	248801591	OR2T35	Exon	C	T
1	248801601	OR2T35	Exon	T	TCA
1	248801609	OR2T35	Exon	G	A
1	248801610	OR2T35	Exon	C	T
2	87088963	CD8B	Exon	A	C
2	118743629	CCDC93	Exon	G	A
2	179528037	TTN	Exon	A	C
3	75790894	ZNF717	Exon	G	GATAGCAGGCTGTTATAAGTCTCCAGCATCACATCCCTGTACAGGGTCCTCTGAGCATTATCCAGGTCCTGCCACTCCTCCCAGGTGAAGTGCACAGCAACATCTTCAAAAGATATCAACCCCTGTAATGGCAT
6	32487424	HLA-DRB5	Splice site	TCAACTA	T
6	32489742	HLA-DRB5	Exon	C	CTG
6	32489744	HLA-DRB5	Exon	CGG	C
6	32489750	HLA-DRB5	Exon	GC	G
6	32489753	HLA-DRB5	Exon	TG	T
6	32489757	HLA-DRB5	Exon	T	TGC
6	32489926	HLA-DRB5	Exon	T	G
6	32489939	HLA-DRB5	Exon	G	C
6	32551960	HLA-DRB1	Exon	G	GT
6	32551961	HLA-DRB1	Exon	C	CT
6	32605265	HLA-DQA1	Exon	G	A
6	52303360	EFHC1	Exon	G	A
6	131456930	AKAP7	Splice site	C	T
7	100647511	MUC12	Exon	C	T
7	138713476	ZC3HAV1L	Exon	C	T
9	134615178	RAPGEF1	Exon	A	G
11	1651227	KRTAP5-5	Exon	C	CTGTGGGGGCTGTGGCTCCGGCTGTGG
11	61725598	BEST1	Splice site	A	ATCCTCCTCC

11	71238674	KRTAP5-7	Exon	C	CCTGCTGCCAGTCCAGCTGCTGTAAGCCCTG
12	57863432	GLI1	Exon	C	T
14	74040135	ACOT2	Exon	G	A
14	105412162	AHNAK2	Exon	C	G
15	30905334	GOLGA8H	Exon	G	C
16	89267936	SLC22A31	Exon	G	A
17	263366	C17orf97	Exon	A	T
17	44408775	LRRC37A	Exon	T	C
17	73596687	MYO15B	Exon	CGGAG	C
17	77078068	ENGASE	Exon	G	A
19	501700	MADCAM1	Exon	G	GACACCACCTCCCCGGAGCCTCCCA
19	501742	MADCAM1	Exon	T	TCTCCCGACACCACCTCCCAGGAGCCTCCCGACACCACCTCCCAGGAGC
19	1088722	POLR2E	Splice site	G	GGAAAGGGGGAGAGTGGTCACAC
19	43376047	PSG1	Exon	T	G
19	43376051	PSG1	Exon	G	C
19	55329850	KIR3DL1	Exon	A	G
20	1592357	SIRPB1	Splice site	C	T

Supplementary Table S2B: List of all compound heterozygous variants (n=116) with allelic frequency of < 0.10 identified in the index patient (single-index WES).

Chr	Position	Gene	Location	Ref	Alt
1	149760094	HNRNPCL1	Exon	C	G
1	12907468	HNRNPCL1	Exon	T	A
1	12907457	HNRNPCL1	Exon	C	A
1	12907517	HNRNPCL1	Exon	T	A
1	12907456	HNRNPCL1	Exon	A	G
1	145368517	NBPF10	Exon	C	T
1	145349627	NBPF10	Exon	A	C
1	148024841	NBPF14	Exon	C	A
1	148024902	NBPF14	Exon	C	T
1	248722755	OR2T29	Exon	T	A
1	248722766	OR2T29	Exon	T	C
1	32541408	TMEM39B	Exon	T	A
1	32541417	TMEM39B	Exon	T	A
6	30997602	MUC22	Exon	CAACACAGCCTGT	C
6	30997621	MUC22	Exon	GGTTCTGAGA	G
6	30997637	MUC22	Exon	CACCCTCCAG	C
6	32497900	HLA-DRB5	Exon	C	G
6	32497912	HLA-DRB5	Exon	C	A

6	32551950	HLA-DRB1	Exon	GCC	G
7	20721256	ABCB5	Exon	C	T
7	20782599	ABCB5	Exon	G	A
7	100550252	MUC3A	Exon	G	T
7	100550280	MUC3A	Exon	C	CTGA
7	100550383	MUC3A	Exon	C	T
7	100551325	MUC3A	Exon	C	CA
7	100551328	MUC3A	Exon	C	CA
7	100551330	MUC3A	Exon	GTA	G
9	33796761	PRSS3	Exon	C	G
9	33796765	PRSS3	Exon	C	T
9	33797927	PRSS3	Exon	G	GCC
9	33797929	PRSS3	Exon	GAC	G
9	33797968	PRSS3	Exon	T	A
9	33797977	PRSS3	Exon	G	A
10	102247437	SEC31B	Exon	C	T
10	102249082	SEC31B	Exon	T	A
11	1260218	MUC5B	Exon	C	T
11	1260216	MUC5B	Exon	C	A
11	1017276	MUC6	Exon	A	G
11	1016933	MUC6	Exon	G	A
11	1016976	MUC6	Exon	G	A
11	1017272	MUC6	Exon	A	G
11	1018472	MUC6	Exon	A	T
11	1018458	MUC6	Exon	G	T
11	1016915	MUC6	Exon	A	G
11	1016967	MUC6	Exon	T	C
11	56143897	OR8U1	Exon	G	T
11	56143995	OR8U1	Exon	A	T
11	56143975	OR8U1	Exon	C	A
11	56143906	OR8U1	Exon	A	G
11	56143986	OR8U1	Exon	G	T
11	56144008	OR8U1	Exon	A	G
11	118499076	PHLDB1	Exon	G	A
11	118526582	PHLDB1	Exon	G	A
12	53343006	KRT18	Exon	G	A
12	53343039	KRT18	Exon	C	A
12	53343104	KRT18	Exon	C	T
12	53343157	KRT18	Exon	A	G
12	53343035	KRT18	Exon	C	T
12	53343032	KRT18	Exon	G	A
12	53343068	KRT18	Exon	G	T
12	53343058	KRT18	Exon	C	A
12	53343098	KRT18	Exon	G	A
12	53343050	KRT18	Exon	G	T
12	11286275	TAS2R30	Exon	G	C

12	11286288	TAS2R30	Exon	A	T
12	11286796	TAS2R30	Exon	A	G
12	11286745	TAS2R30	Exon	A	G
12	11286806	TAS2R30	Exon	T	C
12	11286281	TAS2R30	Exon	G	A
12	11244090	TAS2R43	Exon	C	T
12	11244095	TAS2R43	Exon	T	C
12	11244066	TAS2R43	Exon	A	ATT
12	11244796	TAS2R43	Exon	C	A
12	11244069	TAS2R43	Exon	TCC	T
13	25671331	PABPC3	Exon	CA	C
13	25671273	PABPC3	Exon	C	T
13	25670906	PABPC3	Exon	C	A
13	25671209	PABPC3	Exon	C	T
13	25671213	PABPC3	Exon	T	G
13	25671271	PABPC3	Exon	AG	A
13	25671291	PABPC3	Exon	C	T
13	25670918	PABPC3	Exon	A	G
13	25671026	PABPC3	Exon	A	G
13	25671270	PABPC3	Exon	A	G
13	25670987	PABPC3	Exon	T	G
13	25671309	PABPC3	Exon	TTATGA	T
14	39769121	RP11-407N17.3	Exon	A	C
14	39790148	RP11-407N17.3	Exon	C	G
16	5134778	ALG1	Exon	C	A
16	5122040	ALG1	Exon	C	A
16	29496930	RP11-231C14.4	Exon	ACACACTC	A
16	29496938	RP11-231C14.4	Exon	GGGAGGTGTCTTGAGATTATCATCCGCTGAGGGTGGAAGGGGAGTGAGCAC	G
19	1037780	CNN2	Exon	C	A
19	1037800	CNN2	Exon	A	C
19	1037806	CNN2	Exon	G	A
19	1037809	CNN2	Exon	G	C
19	1037827	CNN2	Exon	A	G
19	1037843	CNN2	Exon	A	G
19	1037855	CNN2	Exon	T	C
19	1037870	CNN2	Exon	C	A
19	8999442	MUC16	Exon	T	C
19	8999496	MUC16	Exon	G	GACCA
19	8999497	MUC16	Exon	GCTTT	G
19	8999511	MUC16	Exon	G	C
19	8999529	MUC16	Exon	C	T
19	8999538	MUC16	Exon	T	C

19	8999553	MUC16	Exon	C	G
19	8999559	MUC16	Exon	T	C
19	9000168	MUC16	Exon	C	T
19	9000186	MUC16	Exon	C	T
19	9000204	MUC16	Exon	C	A
22	37906307	CARD10	Exon	GC	G
22	37906309	CARD10	Exon	TCCTTC	T
X	118605000	SLC25A5	Exon	G	T
X	118605011	SLC25A5	Exon	G	C
X	118605016	SLC25A5	Exon	C	T

Supplementary Table S2C: List of all heterozygous variants (n=127) with allelic frequency of < 0.01 identified in the index patient (single-index WES).

Chr	Position	Gene	Location	Ref	Alt
1	17928622	ARHGEF10L	Exon	G	A
1	64114217	PGM1	Exon	T	G
1	149760094	FCGR1A	Exon	A	C
1	159921649	SLAMF9	Exon	T	C
1	183079677	LAMC1	Exon	G	A
1	197086934	ASPM	Exon	G	T
1	232650123	SIPA1L2	Exon	C	T
2	46707807	TMEM247	Exon	C	CAGCGGCAGCACGAGGTGGTGATGGAGCAGCTGCAGCGGG
2	54117329	PSME4	Exon	C	T
2	55561699	CCDC88A	Exon	G	A
2	128238668	IWS1	Exon	G	C
2	196545571	SLC39A10	Exon	C	T
2	234638244	UGT1A3	Exon	C	G
2	241530238	CAPN10	Exon	C	T
3	15425668	METTL6	Exon	C	T
3	47952109	MAP4	Exon	C	A
3	49051299	WDR6	Exon	G	A
3	128664631	RP11-723O4.6	Exon	G	A
4	1719959	TMEM129	Exon	G	C
4	6578374	MAN2B2	Exon	G	C
4	15713474	BST1	Exon	A	G
4	114276500	ANK2	Exon	A	C
4	159493960	RXFP1	Exon	G	T
5	1243855	SLC6A18	Exon	C	T
5	56168809	MAP3K1	Exon	A	T
5	68550455	CDK7	Exon	T	G
5	68603818	CCDC125	Exon	C	T
5	95067850	RHOBTB3	Exon	CT	C

6	24357924	DCDC2	Exon	G	T
6	35428347	FANCE	Exon	T	C
6	36446921	KCTD20	Exon	C	A
6	52268519	PAQR8	Exon	C	T
6	88853810	CNR1	Exon	T	C
6	111634582	REV3L	Exon	C	T
6	155728366	NOX3	Exon	A	T
7	4800901	FOXK1	Exon	T	C
7	134730585	AGBL3	Exon	A	G
7	155532702	RBM33	Exon	C	T
8	12044036	FAM86B1	Exon	G	C
8	12863754	KIAA1456	Exon	A	G
7	33345938	MAK16	Exon	A	C
8	101164188	POLR2K	Exon	G	T
8	110523157	PKHD1L1	Exon	A	C
8	113314102	CSMD3	Exon	A	C
8	2646341	VLDLR	Exon	T	C
9	90220083	DAPK1	Exon	T	G
9	123874802	CNTRL	Exon	G	T
9	130475065	C9orf117	Exon	G	A
9	5922265	ANKRD16	Exon	C	A
10	71905755	TYSND1	Exon	T	TC
10	75407884	SYNPO2L	Exon	AG	A
10	97115623	SORBS1	Exon	G	A
10	10874822	ZBED5	Exon	C	T
11	45928492	C11orf94	Exon	C	G
11	62996801	SLC22A25	Exon	G	A
11	65409055	SIPA1	Exon	T	C
11	66031179	KLC2	Exon	G	A
11	72004487	CLPB	Exon	TG	T
11	119053025	NLRX1	Exon	C	T
11	125791184	DDX25	Exon	G	A
12	2027515	CACNA2D4	Exon	C	G
12	45610173	ANO6	Exon	G	A
12	48596358	OR10AD1	Exon	GAAAGTCT TC	G
12	56504429	PA2G4	Exon	T	C
12	58112038	OS9	Exon	AGAGGAG	A
12	89819155	POC1B	Exon	T	C
12	113532942	DTX1	Exon	T	C
12	23929531	SACS	Exon	G	T
13	45554905	NUFIP1	Exon	C	T
13	20002295	POTEM	Exon	C	A
14	21167839	RNASE4	Exon	G	A
14	24799484	ADCY4	Exon	C	T
14	36004143	INSM2	Exon	A	G
14	22873209	TUBGCP5	Exon	C	G

15	51792371	DMXL2	Exon	T	C
15	52567760	MYO5C	Exon	C	T
15	59445836	MYO1E	Exon	G	A
15	338165	AXIN1	Exon	C	T
16	5134778	ALG1	Exon	C	A
16	11792036	TXNDC11	Exon	G	T
16	67701397	C16orf86	Exon	C	G
16	70916786	HYDIN	Exon	C	T
16	8025157	HES7	Exon	C	A
17	40376873	STAT5B	Exon	G	A
17	45925073	SP6	Exon	G	A
17	18276980	PIK3R2	Exon	G	T
19	21991118	ZNF43	Exon	C	G
19	33499031	RHPN2	Exon	C	G
19	38964160	RYR1	Exon	G	C
19	42776511	CIC	Exon	A	T
19	49377557	PPP1R15A	Exon	GGAA	G
19	49983683	FLT3LG	Exon	G	A
19	52868950	ZNF610	Exon	G	T
19	55870686	FAM71E2	Exon	G	C
19	5903234	CHGB	Exon	T	C
20	25472060	NINL	Exon	C	A
20	36992703	LBP	Exon	T	A
20	46032444	KRTAP10-8	Exon	C	T
21	29085194	CHEK2	Exon	G	T
22	46657498	PKDREJ	Exon	A	G
22	51009933	CPT1B	Exon	G	A
22	19026116	GPR64	Exon	T	A
X	37699832	DYNLT3	Exon	G	T
X	68382551	PJA1	Exon	T	G
X	84362361	SATL1	Exon	T	C
X	86067885	DACH2	Exon	T	C
X	128957717	ZDHHC9	Exon	A	G
X	150912763	CNGA2	Exon	G	A
X	153039548	PLXNB3	Exon	T	C
X	154344992	BRCC3	Exon	C	T
X	2418256	DHRSX	Exon	T	TC
X	2861391	ARSE	Exon	AT	A
X	11133167	HCCS	Exon	C	A
X	16711387	CTPS2	Exon	C	A
X	18602328	CDKL5	Exon	A	G
X	19026116	GPR64	Exon	T	A
X	24091400	EIF2S3	Exon	C	T
X	49218226	GAGE12I	Exon	C	A
X	69718532	DLG3	Exon	CTCAT	C
X	70673947	TAF1	Exon	C	T

X	70788114	OGT	Exon	A	G
X	108684441	GUCY2F	Exon	C	T
X	134986780	SAGE1	Exon	G	GTATGTC
X	140993820	MAGEC1	Exon	T	TTGAGTATTTTCCAGAGTTCCCCTGAGAGAACTCAGAGTACTTTTGAGGGTTTTGCCCAGTCTCCACTCCAGATTCCTGTGAGCCCCTCCTTCTCCTCCACTTTAG
X	153070431	PDZD4	Exon	A	C
X	153482562	TEX28P1	Exon	G	C

Supplementary Figure S1: Cluster identification according to the expression of major markers and traditional flow cytometry.

a-b) Density histograms showing the expression level of all considered markers in each FlowSOM cluster of a) CD14-CD3+CD4+ T cells and b) CD14-CD3+CD8+ T cells. c-d) Quantification of all the cell population clusters identified within c) *CD14-CD3+CD4+ T cells and d) CD14-CD3+CD8+ T cells.* One independent experiment (2 technical repeats) with 3 HCs. Values presented as the mean + SEM. *e-f) Heatmap of all the flow cytometry markers used for the flowSOM clustering of e) CD14-CD3+CD4+ T cells and f) CD14-CD3+CD8+ T cells for each control and patient replicate.*

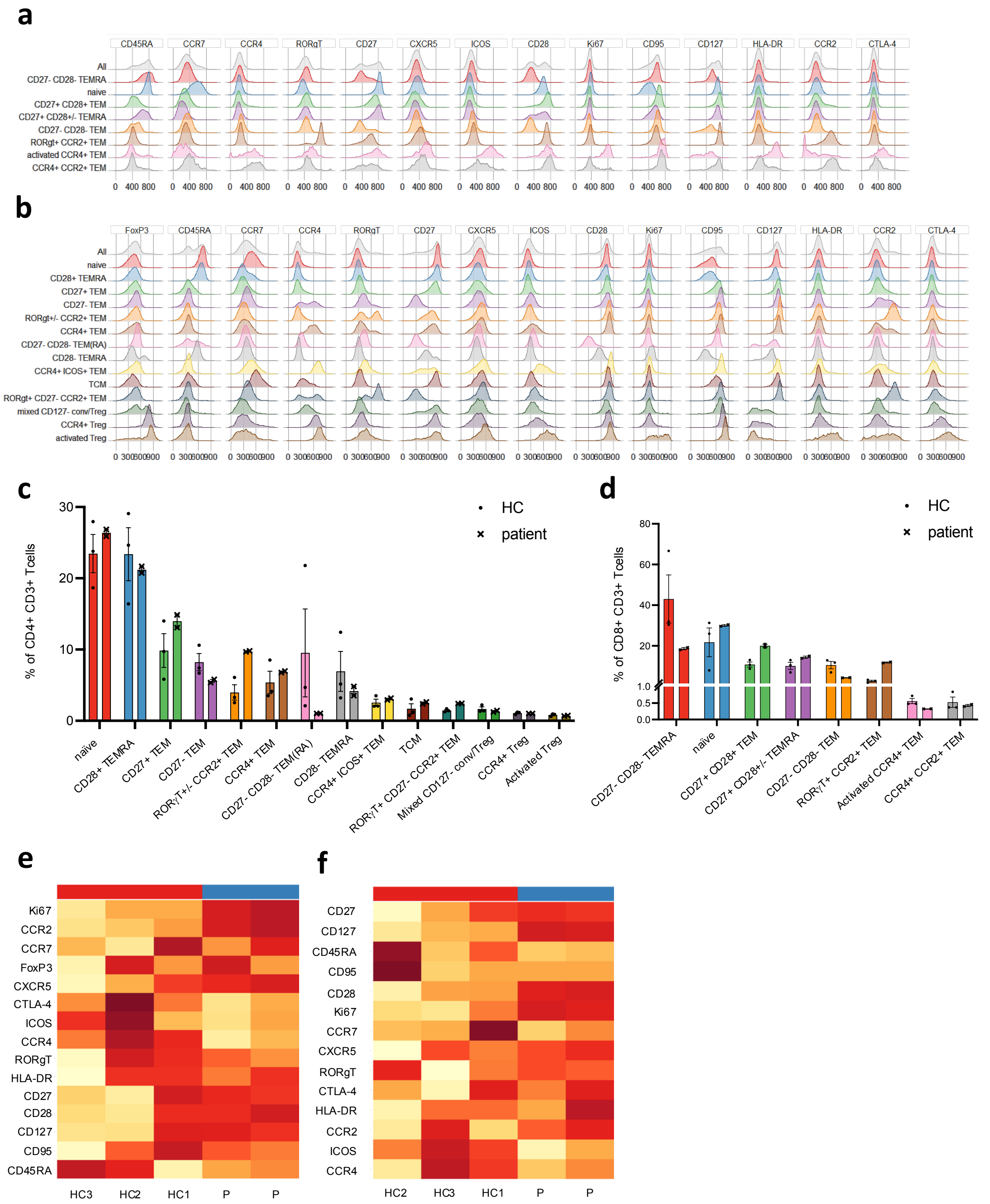
a
CD45RA
CCR7
CCR4
RORgT
CD27
CXCR5
ICOS
CD28
Ki67
CD95
CD127
HLA-DR
CCR2
CTLA-4
All
CD27- CD28- TEMRA
naive
CD27+ CD28+ TEM
CD27+ CD28+/- TEMRA
CD27- CD28- TEM
RORgt+ CCR2+ TEM
activated CCR4+ TEM
CCR4+ CCR2+ TEM
0 400 800
b
FoxP3
CD45RA
CCR7
CCR4
RORgT
CD27
CXCR5
ICOS
CD28
Ki67
CD95
CD127
HLA-DR
CCR2
CTLA-4
All
naive
CD28+ TEMRA
CD27+ TEM
CD27- TEM
RORgt+/- CCR2+ TEM
CCR4+ TEM
CD27- CD28- TEM(RA)
CD28- TEMRA
CCR4+ ICOS+ TEM
TCM
RORgt+ CD27- CCR2+ TEM
mixed CD127- conv/Treg
CCR4+ Treg
activated Treg
0 300 600 900
c
HC
patient
% of CD4+ CD3+ Tcells
0
10
20
30
naive
CD28+ TEMRA
CD27+ TEM
CD27- TEM
RORγT+/- CCR2+ TEM
CCR4+ TEM
CD27- CD28- TEM(RA)
CD28- TEMRA
CCR4+ ICOS+ TEM
TCM
RORγT+ CD27- CCR2+ TEM
Mixed CD127- conv/Treg
CCR4+ Treg
Activated Treg
d
HC
patient
% of CD8+ CD3+ Tcells
0.0
0.5
1.0
20
40
60
80
CD27- CD28- TEMRA
naive
CD27+ CD28+ TEM
CD27+ CD28+/- TEMRA
CD27- CD28- TEM
RORγT+ CCR2+ TEM
Activated CCR4+ TEM
CCR4+ CCR2+ TEM
e
Ki67
CCR2
CCR7
FoxP3
CXCR5
CTLA-4
ICOS
CCR4
RORgT
HLA-DR
CD27
CD28
CD127
CD95
CD45RA
HC3
HC2
HC1
P
P
f
CD27
CD127
CD45RA
CD95
CD28
Ki67
CCR7
CXCR5
RORgT
CTLA-4
HLA-DR
CCR2
ICOS
CCR4
HC2
HC3
HC1
P
P

Supplementary Figure S2: Flow gating strategy for IL-17 production by CD4+ T cells after anti-CD3/CD28 and IL-23 stimulation.

The complete gating strategy for IL-17 production by CD4+ T cells after stimulation with anti CD3/CD28 and IL-23 is shown for HC. The final % of IL-17 + CD4+ T cells is shown for two HC, Patient (P), Brother (B), Father (F) and mother (M).

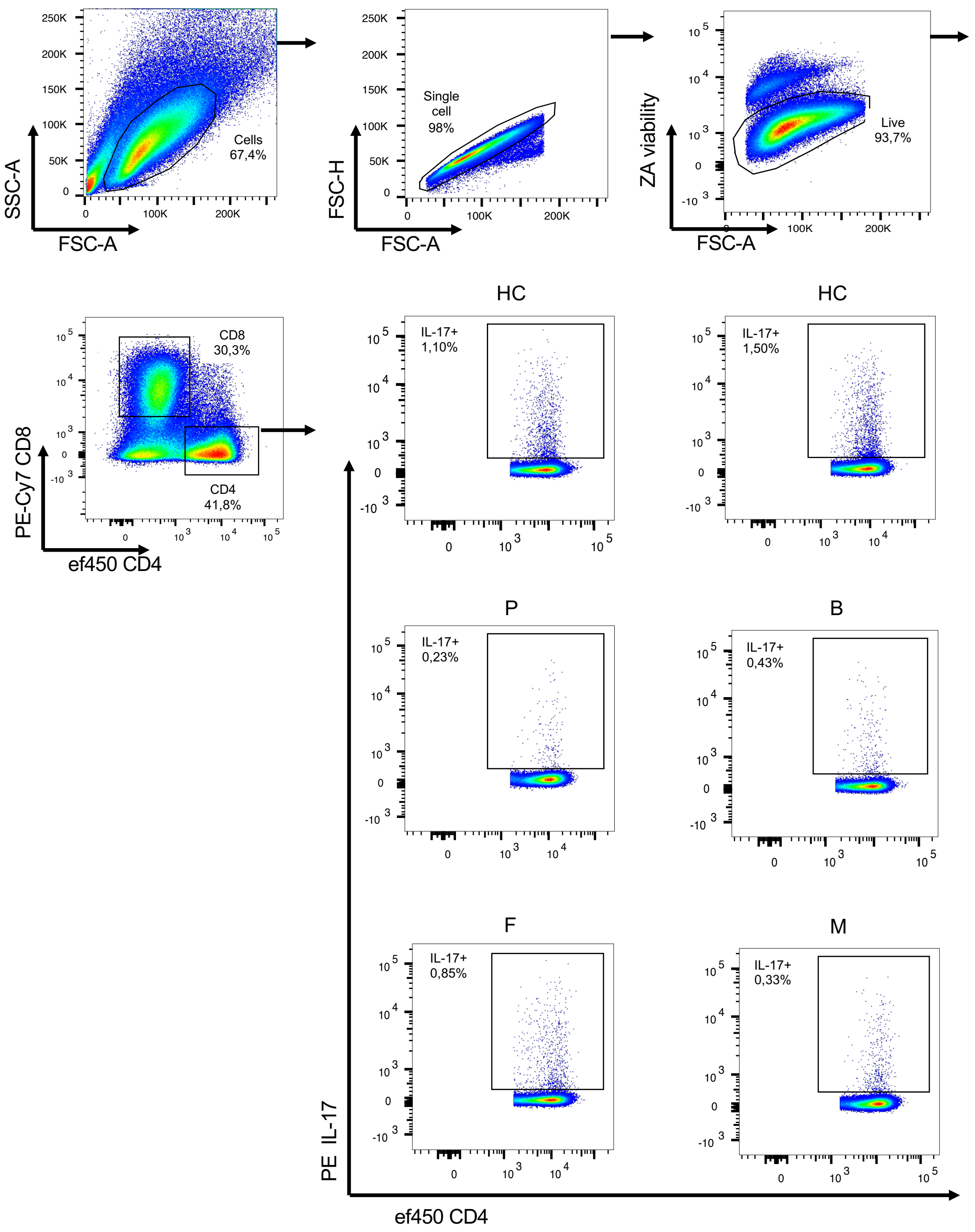

Supplementary Figure S3: Flow gating strategy for STAT3 phosphorylation in response to IL-23 stimulation in primary cells of R381Q homozygous carrier.

The complete gating strategy for pSTAT3 within CD4+ T and CD8+ T cells after stimulation with IL-23, IL-1b or IL-6 is shown for HC. The final % of pSTAT3 within CD4+ or CD8+ T cells is shown for HC and Patient (P)

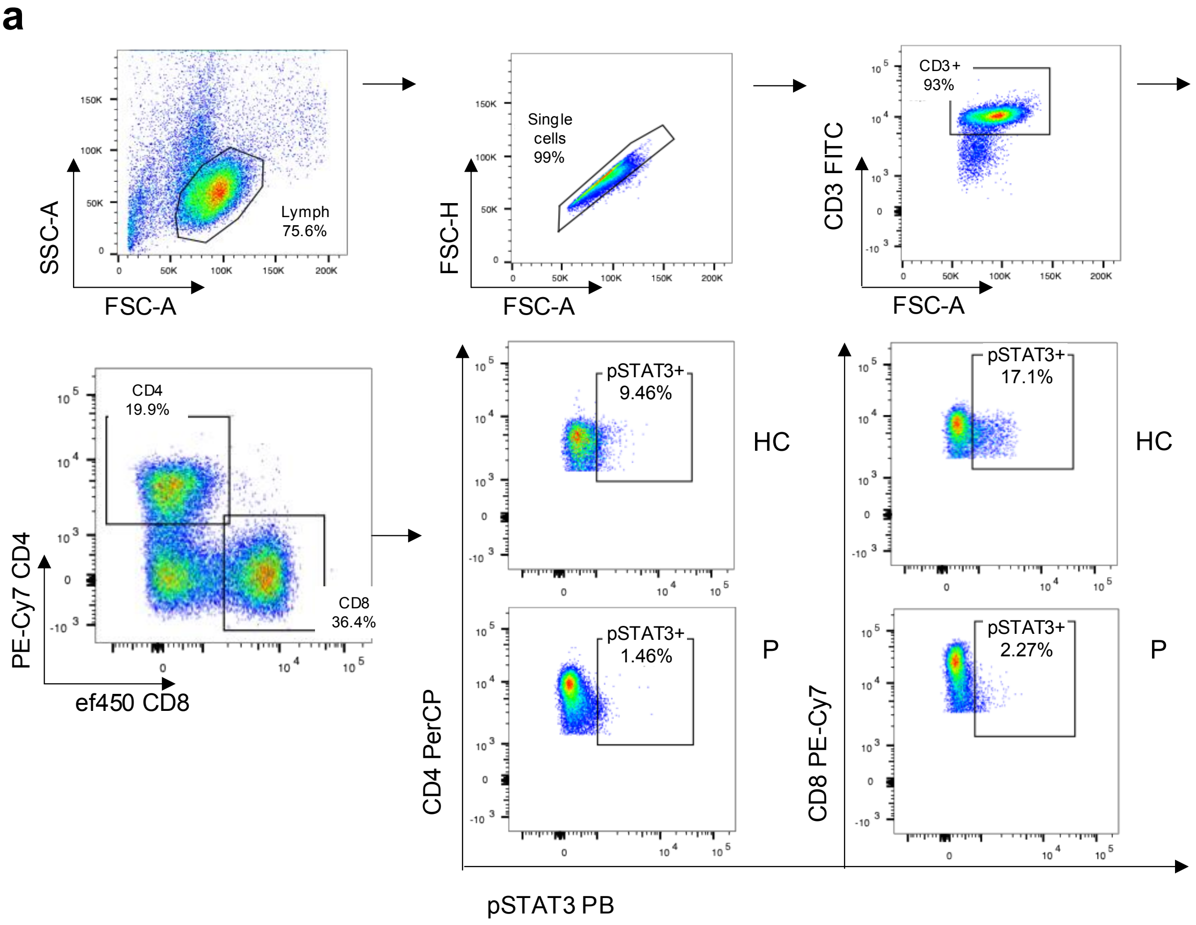

Supplement: Supplementary file 1 — Supporting Information file 1: eji70002‐sup‐0001‐SuppMat.pdf [file EJI-55-e70002-s001.pdf]
